# The Treatment of Joints in Rheumatoid Arthritis

**Published:** 1907-05-04

**Authors:** James Lindsay


### The General Practitioners' Column.

### THE TREATMENT OF JOINTS IN RHEUMATOID ARTHRITIS.

By JAMES LINDSAY, M.D.Edin.

The condition of rheumatoid arthritis, on account of the urgency of the symptoms and the progressive nature of the disease, is one which calls for the most careful consideration from the general practitioner. In the acute poly-articular form of the disease, met with mostly in children and young adults, the medicinal treatment is on much the same lines as that in acute rheumatism—namely, the employment of salicylates. In these, as also in the more chronic forms, there should be a careful examination of the various orifices for purulent discharges—*e.g.* otorrhœa, leucorrhœa, etc.; also of the skin surface for ulcerations or abscesses, which must be attended to without delay.

### Local Measures.

Locally, in the acute form, when the joints are painful, swollen, and inflamed, much relief is afforded by fixing the joint with a splint. At the same time relief from pain is afforded by applying lin. A.B.C. on a piece of lint over the joint, or by the frequent application, every two hours or so, of carbolic acid fomentations—equal parts of 1 to 20 solution with hot water. Following this, a paint which consists of—

Guaiacol ... ... ... ... ... 1 part
Tinct. iodi ... ... ... ... 6 parts may be applied twice daily over the affected joint, often giving the most satisfactory results.

### Intestinal Antiseptics.

In the chronic poly-articular form the most satisfactory medicinal treatment consists in the administration of salol, guaiacol carbonate and $\beta$ naphthol. In order to explain their action one has only to accept the bacterial or more probably the auto-toxic theory of the causation of the disease. *Salol,* a drug of great value in this condition, may be prescribed either alone or in combination with guaiacol or salicylate of quinine. In the intestine it breaks up into its component parts, salicylic and carbolic acids, which act directly on the bacteria and their toxins. In the form of a mixture salol may be prescribed thus:—

℞ Salol ... ... ... ... ... ... gr. vj.
Quin. salicyl. ... ... ... ... gr. vj.
Pulv. trag. co. ... ... ... ... gr. x.
Aquam ad ... ... ... ... ... ℥j.
T.d.s.

*Guaiacol carbonate* also gives highly satisfactory results. It has the advantage over guaiacol in not producing any symptoms of gastric discomfort. Its administration may be carried on over a prolonged period—a very essential point in the treatment of a chronic disease like this. It is usually prescribed in cachets, gr. v.-x. t.i.d., or in the following mixture:—

℞ Guaiacol carb. ... ... ... ... gr. x.

Mucil. tragac. ... ... ... ... ʒij.

Aquam ad ... ... ... ... ... ℥j.

T.d.s.

$\beta$ *Naphthol* is best prescribed as a pill:—

℞ $\beta$ naphthol. ... ... ... ... gr. iij.

Pulv. tragac. ... ... ... } aa. q.s.

Syr. simp. ... ... ... ... }

Ft. pil. t.d.s.

For the anæmia which is so frequently associated with the condition as to constitute a symptom, preparations of iron are indicated. The most usual form is a pil. Blaud gr. v. t.i.d., or as the saccharated carbonate of iron gr. x. in cachets t.i.d.—a form which appears to act particularly well in these cases.

### Further Points in Treatment.

Locally the painful joints may be painted with the tincture of iodine. In the more chronic types the application of paints containing salicylate of methyl and guaiacol is followed by considerable alleviation of joint pain. Such preparations as these have been found most suitable:—

℞ Methyl. salicyl. ... ... ... ... 1 part

Ol. olivæ ... ... ... ... ... 2 parts

Ft. pigm.

Guaiacol, ... ... ... ... } aa. æq. partes

Ol. olivæ ... ... ... ... }

Ft. pigm.

Blistering painful joints is of much value, particularly in the larger joints—hips, knees, ankles, elbows, and back of wrists. To the raw surface a protective dressing of a simple nature, as 1-7 boric acid with vaseline, should be applied.

In the further local treatment of the joints in rheumatoid arthritis, active and passive movements should be carried out so as to prevent the formation of adhesions with stiffening and ankylosis. Galvanism is also of great use.

It will be observed that the atrophy of muscles is often very considerable even in the early stages, and in addition that it is confined to particular groups of muscles, thus suggesting a nerve lesion rather than mere disuse as the cause.

This being so, care should be exercised in massaging the part to concentrate one's efforts on such groups of muscles, or even individual muscles. In massaging, a mixture of equal parts of camphorated oil and soap liniment may be used as a lubricant, or instead powdered starch may be used, which is certainly more cleanly and pleasant to work with.

The weak constant current will do much to improve the nutrition of the muscles in connection with the affected joints. An ordinary battery, possessing from 10 to 40 Leclanché cells, may be used. The pole is applied over the muscle; if in the lower limb the + pole in the form of a flat pad being placed over the lumbar region; while if in the upper limb it is placed over the cervical region of the spine.

